# Quality of life in pre- and postmenopausal patients with early breast cancer: a comprehensive analysis from the prospective MaLife project

**DOI:** 10.1007/s10549-019-05197-w

**Published:** 2019-03-13

**Authors:** Norbert Marschner, Tanja Trarbach, Jacqueline Rauh, Dirk Meyer, Sigrun Müller-Hagen, Johanna Harde, Stephanie Dille, Lisa Kruggel, Martina Jänicke, The TMK-Group (Tumour Registry Breast Cancer)

**Affiliations:** 1 Praxis für interdisziplinäre Onkologie und Hämatologie, Wirthstrasse 11c, 79110 Freiburg, Germany; 2 MVZ des Klinikums Wilhelmshaven, Zentrum für Tumorbiologie, Wilhelmshaven, Germany; 3 GIM Gemeinschaftspraxis Innere Medizin, Witten, Germany; 4 OSP Göttingen, Göttingen, Germany; 5 Hämatologisch-Onkologischer Schwerpunkt Schnelsen, Hamburg, Germany; 6 Department of Biostatistics, iOMEDICO, Freiburg, Germany; 7 Medical Department, iOMEDICO, Freiburg, Germany; 8 Clinical Epidemiology and Health Economics, iOMEDICO, Freiburg, Germany

**Keywords:** Breast neoplasms, Registries, Cohort studies, Quality of life, Outpatients, Questionnaires, Menopause

## Abstract

**Purpose:**

Quality of life (QoL) plays an important role in recovery—especially after an incisive diagnosis such as breast cancer. Here, we present a comprehensive assessment of QoL for pre- and postmenopausal patients, starting from initial systemic treatment of early breast cancer until 3 years later, in patients from a so-called “real-world” setting.

**Methods:**

251 premenopausal and 478 postmenopausal patients with early breast cancer have been recruited into the longitudinal MaLife project within the prospective, multicentre, German Tumour Registry Breast Cancer between 2011 and 2015. The questionnaires FACT-G, FACT-Taxane, FACT-ES, EORTC QLQ-BR23, BFI and HADS were filled in at start of treatment (T0), 6, 12, 24 and 36 months later. The proportion of patients with clinically meaningful changes at 36 months was determined.

**Results:**

This first interim analysis shows that the FACT-G global QoL improved over time regardless of the menopausal status. However, clinically meaningful decrease of social/family well-being (48–51%), arm symptoms (44–49%) and symptoms of neurotoxicity (55–56%) was frequently reported 3 years after start of treatment. Many premenopausal patients also reported a clinically meaningful worsening of endocrine symptoms (64%), emotional well-being (36%) and fatigue intensity (37%). Additionally, 3 years after start of treatment, 15% of the patients were classified as doubtful cases and 18% as definite cases of anxiety.

**Conclusions:**

Despite improvements in global QoL, breast cancer survivors report worsened ailments 3 years after start of therapy. Follow-up care should distinguish between premenopausal patients needing special attention for emotional/menopausal issues, and postmenopausal patients needing particular care regarding physical concerns.

**Electronic supplementary material:**

The online version of this article (10.1007/s10549-019-05197-whttps://doi.org/10.1007/s10549-019-05197-w) contains supplementary material, which is available to authorized users.

## Introduction

Breast cancer remains the most frequent cancer among women, with approximately 266,000 estimated new cases in the US and 72,000 in Germany in 2018 [1, 2]. With a 5-year survival rate of almost 90%, there is a growing number of breast cancer survivors in need of optimal care [1, 2]. In 2015, their number reached more than 3.4 million in the United States alone [2]. In patients with early breast cancer, standard of care is the surgical removal of the tumour, preceded or followed by chemotherapy, and/or preceded by radiation therapy depending on the individual risk profile of the patient. The majority (approximately 75%) of breast cancer cases are hormone receptor-positive (HR-positive) tumours [3] for whom additional adjuvant endocrine treatment is recommended.

The choice of the specific endocrine treatment depends on the menopausal status of the patient. Premenopausal patients should receive adjuvant tamoxifen and optionally ovarian suppression, while postmenopausal patients should receive an aromatase inhibitor (anastrozole, letrozole, exemestane) or tamoxifen followed by an aromatase inhibitor [reviewed in 4]. Treatment with either tamoxifen or aromatase inhibitors should last for at least 5 years, thus the patients have to deal with a long period of medical interventions, potential side effects and the associated psychological strain, all of which can strongly affect the quality of life (QoL) [5–7].

Although recent findings suggest that endocrine therapy alone is adequate for women with low-risk tumours [8, 9], additional adjuvant chemotherapy is widely used. Both systemic treatment options are associated with side effects and toxicities: while endocrine therapy may cause osteopenia, osteoporosis, arthralgia, musculoskeletal symptoms and menopausal complaints [10–14]; chemotherapy is associated with nausea, hair loss, paraesthesia, neuropathy and cardiotoxicity, some of which are persistent even years after treatment [15–17]. Furthermore, crucial side effects for premenopausal patients are treatment-induced amenorrhea or infertility [18–21]. Indeed, it has been published that younger women report greater changes for the worse regarding mood and emotional functioning in comparison to older women [22, 23].

Of note, QoL is not only impaired by side effects, but also by other factors like lack of social support or expenditure of time for treatment. These factors are especially troubling for young patients, mostly focused on career and/or caring for young children. Balancing the efficacy of systemic treatments with upholding QoL for the patient is one of the biggest challenges for oncologists. Broad and detailed evaluations of patient-reported outcomes (PROs) are necessary in order to highlight all short- and long-term effects of treatment, especially because of the growing number of breast cancer patients treated with curative intent.

The clinical cohort study TMK (Tumour Registry Breast Cancer) set out to examine the treatment of early and metastatic breast cancer in German routine care as well as the impact of the disease on various aspects of life. For patients with early breast cancer, we previously published data on the routine treatment with adjuvant chemotherapy [24] and results from the MaTox project on toxicity-related symptoms after adjuvant chemotherapy [15]. In the analysis at hand, we present data from the longitudinal MaLife project—a comprehensive assessment of PROs in a real-world setting, aiming to get a detailed understanding of the situation of patients with breast cancer in German routine care. We looked at patients of all breast cancer subtypes with documented pre- or postmenopausal status and compared the temporal change in QoL during systemic treatment and over the following 3 years.

## Patients and methods

### Data source

The TMK is an ongoing, open, longitudinal, multicentre, observational, prospective cohort study which started in 2007. The study was approved by the responsible ethics committee and is registered at ClinicalTrials.gov (NCT01351584). Eligible patients are women aged ≥ 18 years with histologically confirmed breast cancer and starting systemic antineoplastic treatment. Written informed consent is obtained from all patients. Enrolment is restricted to patients who sign informed consent no longer than 6 weeks after start of treatment. The TMK has previously been described in detail [25].

The MaLife project is an ongoing, prospective, longitudinal survey within the TMK that recruited patients with early and advanced breast cancer between 2011 and 2015 to evaluate PROs during and after systemic treatment.

### Cohort definition

Until data cut at 31, October 2017, a total of  2013 patients had been recruited for the MaLife project. Out of these, 1014 patients were recruited in neoadjuvant or adjuvant treatment intention. Of these, 137 patients were excluded as they had not sent back a single questionnaire or had incomplete basic medical data. 61 patients were excluded because they were classified as ‘perimenopausal’ at inclusion and 87 patients were excluded due to unknown menopausal status. The present analysis focused on 729 patients receiving neoadjuvant or adjuvant therapy, 251 with premenopausal, and 478 with postmenopausal status at start of therapy. The patients were recruited by 98 sites of office- and hospital-based medical oncologists and gynaecologists located all over Germany.

### Questionnaires

The specifically compiled MaLife questionnaire encompasses 130 items combining both validated instruments as well as additional, specifically designed questions to assess QoL, symptoms associated with breast cancer and its treatment, type and time of health-care resources used, ability to work and other aspects of everyday life impairment. Feasibility of the MaLife questionnaire was tested before the start of the study. The questionnaire was sent to patients by post at start of systemic treatment as well as 6 and 12 months later and then annually up to 5 years. Reminders were sent by post 2 weeks after mailing of the questionnaires and a second reminder another 2 weeks later, if the questionnaire had not yet been returned.

In this interim analysis, we included the results from six validated questionnaires within the MaLife questionnaire: FACT-G for general quality of life [26], the FACT-Taxane subscale for neurotoxicity symptoms, the endocrine symptoms subscale ESS-18 of the FACT-ES [27] and the EORTC QLQ-BR23 for breast cancer-specific symptoms [28]. Additionally, we analysed the results from the Brief Fatigue Inventory, BFI [29] and the Hospital Anxiety and Depression Scale, HADS [30].

### Statistical analysis

Scoring of the questionnaires was performed according to the respective manuals. Mean change to baseline was calculated as the difference between the scores 3 years after start of treatment and the scores at baseline. Clinical meaningful differences for the respective scales were derived from previously established meaningful differences: ≥ 4 points for the FACT-G total score, ≥ 2 points for the FACT-G subscales [31] and 10 points (10% of the instrument range) for the EORTC QLQ BR-23 [32, 33]. For the other scales without previously established meaningful differences, we considered a change equal to or greater than ½ of the standard deviation (SD) at T0 clinically meaningful. This method was previously applied for the BFI and the respective subscales [34] and has been published to be a valid threshold of discrimination for changes in various QoL instruments [35, 36]. For the Taxane subscale of the FACT-Taxane, a change of ≥ 3 points (SD at T0: 5.59) was considered clinically meaningful for the premenopausal patients, and a change of ≥ 4 points (SD at T0: 7.82) for the postmenopausal patients. Accordingly, a change of ≥ 4 points was considered clinically meaningful for the endocrine symptom subscale of the FACT-ES; a change of ≥ 1.2 points for the BFI total score; a change of ≥ 1.3 points on the Fatigue interference score for the premenopausal and a change of ≥ 1.2 points for the postmenopausal patients and a change of ≥ 1.2 points on the Fatigue intensity score. For the HADS, the developers defined three ranges for each subscale and these were implemented to identify the percentage of patients exceeding the cut-off scores: 0–7 (non-cases), 8–10 (doubtful cases) and 11–21 (definite cases) of anxiety/depression [30]. If patients experienced a recurrence of breast cancer during the observation period, the respective questionnaires after such a diagnosis were excluded from the present analysis. All analyses were performed using Dell, Inc. (2016), Dell Statistica (Software-System für Datenanalyse), version 13. software.dell.com.

## Results

### Patient, tumour and treatment characteristics

Basic demographic and clinical data of the pre- and postmenopausal patients receiving neoadjuvant or adjuvant therapy are shown in Table 1. Median age at start of systemic therapy was 45 years for premenopausal and 63 years for postmenopausal patients. 45 of the patients (18%) were aged 50–55 in the premenopausal group, and 71 of the patients (15%) were aged 50–55 in the postmenopausal group. The prevalence of comorbidities was higher for postmenopausal than for premenopausal patients, especially hypertension was documented more frequently (40% of postmenopausal, 9% of premenopausal patients). Regarding receptor status as well as tumour stage at diagnosis, the two subgroups were comparable.

**Table 1:** Patient and tumour characteristics

Characteristic	Premenopausal (*n* = 251)		Postmenopausal (*n* = 478)	
	**Years**	**Min–max**	**Years**	**Min–max**
Median age at start of systemic therapy	45.1	23.4–69.4	62.8	37.6–84.0
	**Mean**	**SD**	**Mean**	**SD**
BMI at enrolment, kg/m^2^	25.5	5.04	27.5	5.57
Patients with comorbidity at diagnosis	***n***	**%**	***n***	**%**
Any comorbidity^a^	95	37.8	335	70.1
CCI = 0^b^	232	92.4	412	86.2
CCI ≥ 1^b^	19	7.6	66	13.8
Hypertension	23	9.2	193	40.4
Diabetes	1	0.4	50	10.5
Cardiovascular disorders	5	2.0	25	5.2
Receptor status at diagnosis				
HR-positive, HER2-negative	158	62.9	279	58.4
HR-positive, HER2-positive	41	16.3	72	15.1
HR-negative, HER2-positive	12	4.8	37	7.7
Triple negative	38	15.1	80	16.7
Unknown*	2	0.8	10	2.1
Tumour stage at diagnosis^c^				
I	60	23.9	134	28.0
II	119	47.4	218	45.6
III	39	15.5	77	16.1
Not determined/Unknown^d^	33	13.2	49	10.2
Nodal stage at diagnosis				
Positive	120	47.8	209	43.7
Negative (N0)	121	48.2	260	54.4
Unknown (NX + missing)	10	4.0	9	1.9

*BMI* Body Mass Index, *HR* hormone receptor, *HER2* human epidermal growth factor receptor 2, *Max* maximum, *Min* minimum, *SD* standard deviation

*This category includes three patients with HR-positive tumours and unknown HER2-status

^a^Comorbidity according to Charlson [48] or additional concomitant diseases

^b^Charlson Comorbidity Index (CCI) according to Quan [49]

^c^Tumour stage according to AJCC/UICC 7th edition

^d^For some patients the exact stage could not be determined because of unknown parameters (TX, NX, MX)

Independent of the menopausal status, most patients underwent a breast-conserving surgery (69%) and 82% received radiotherapy (Table 2). Slightly more postmenopausal women were enrolled at start of adjuvant treatment (84 vs. 71%), while slightly more premenopausal women were enrolled at start of neoadjuvant treatment (29 vs. 16%). 96% of the premenopausal and 90% of the postmenopausal patients initially received chemotherapy, mostly a combination therapy of anthracycline and taxane (Table 2). For 91% of the premenopausal and 82% of the postmenopausal patients with HER2-positive tumours, an additional anti-HER2 therapy with trastuzumab was documented. The same proportion of premenopausal and postmenopausal patients with HR-positive tumours received endocrine therapy (83–84%). 66% premenopausal patients received an oestrogen-receptor antagonists (mostly tamoxifen) compared to 25% postmenopausal patients (Table 2). 42% of the postmenopausal patients received aromatase inhibitors. A switch of endocrine agent was documented in 11% of the premenopausal and 16% of the postmenopausal patients.

**Table 2:** Treatment characteristics

Treatment	Premenopausal (*n* = 251)		Postmenopausal (*n* = 478)	
	*n*	%	*n*	%
Resection of primary tumour				
Breast-conserving (incl. follow-up resection)	173	68.9	332	69.5
Non-breast conserving (mastectomy/ablatio mammae)	56	22.3	114	23.8
Unknown	22	8.8	32	6.7
Radiotherapy				
Yes	205	81.7	392	82.0
No	46	18.3	86	18.0
Therapy setting at enrolment				
Neoadjuvant	72	28.7	79	16.5
Adjuvant	179	71.3	399	83.5
Chemotherapy				
Yes	242	96.4	432	90.4
No	9	3.6	46	9.6
Chemotherapy regimen				
E/A + C + P	70	27.9	105	22.0
E/A + C + D	46	18.3	67	14.0
F + E/A + C + D	40	15.9	66	13.8
F + E/A + C	22	8.8	46	9.6
Others	64	25.5	148	31.0

Treatment	Premenopausal, HR-positive (*n* = 199)		Postmenopausal, HR-positive (*n* = 354)	
	*n*	%	*n*	%
Endocrine therapy				
Yes	166	83.4	296	83.6
No	33	16.6	58	16.4
Endocrine therapy regimen				
Aromatase inhibitors (AI) ± GnRH	12	6.0	149	42.1
Oestrogen-receptor antagonist	132	66.3	88	24.9
Switch ER-antagonist/AI	22	11.1	55	15.5
Others	–	–	4	1.1

*AI* aromatase inhibitor, *C* cyclophosphamide, *D* docetaxel, *E*/*A* epirubicin/doxorubicin, *ER-antagonist* oestrogen-receptor antagonist, *F* fluorouracil, *GnRH* gonadotropin-releasing hormone, *P* paclitaxel

### Questionnaire return rate

**Fig. 1:**
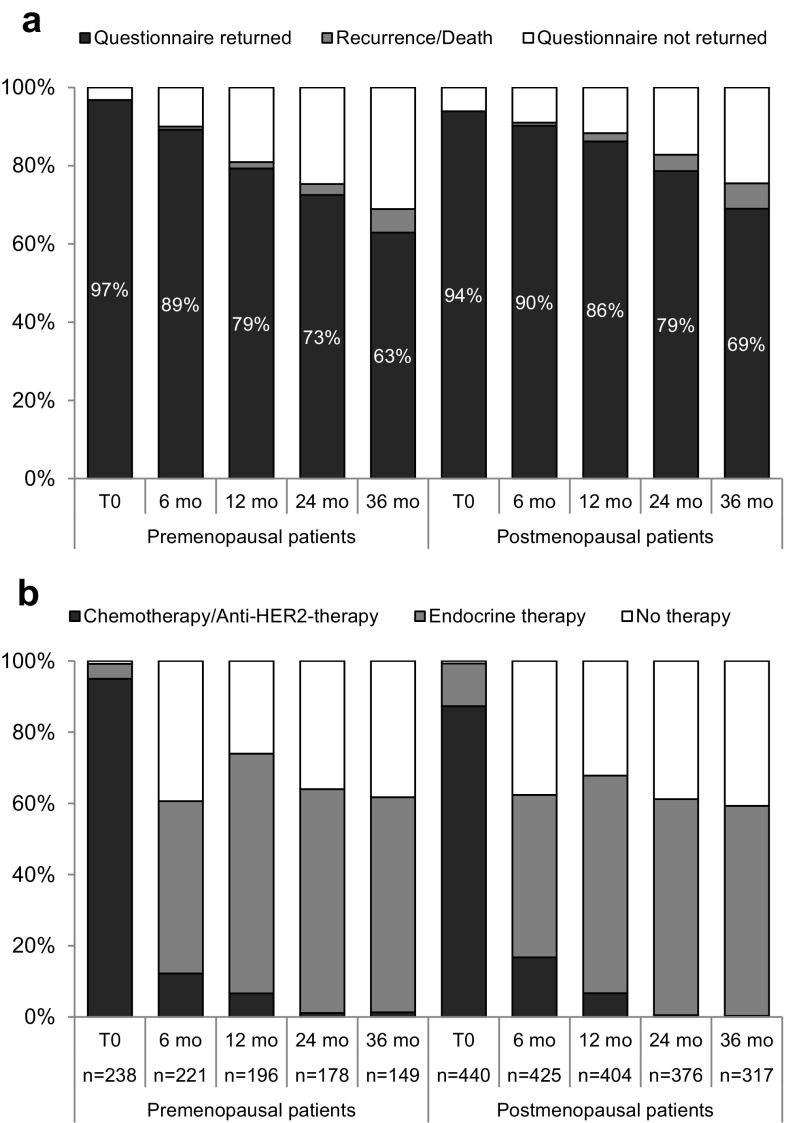
Questionnaire return rate and treatment. **a** Return rate of the MaLife questionnaire for the premenopausal and postmenopausal patients at start of therapy (T0), 6 months, 12 months, 24 months and 36 months later. **b** Proportion of patients receiving systemic chemotherapy and/or anti-HER2-therapy, endocrine therapy or no therapy at the respective questionnaire time points. *mo* months

Return rates for the questionnaire at start of systemic treatment (T0) as well as at all other time points are depicted in Fig. 1a. At later time points, return rates were slightly higher for postmenopausal patients. During the observation period, 16 (6.4%) premenopausal and 49 (10.3%) postmenopausal patients experienced a recurrence; 1 premenopausal patient (0.4%) and 7 postmenopausal patients (1.5%) died.

Looking at the treatment at the respective questionnaire time points, 95% (87%) of the premenopausal (postmenopausal) patients started systemic chemotherapy and/or anti-HER2-therapy at T0 and this number declined to 12% (17%) 6 months and 7% (7%) 12 months later (Fig. 1b). Overall, approximately 60% of all patients (both HR-positive and -negative) received endocrine therapy at 12, 24 and 36 months, respectively.

### QoL and symptom severity at start of treatment

Baseline mean values at start of treatment (T0) for FACT-subscales and HADS were similar for pre- and postmenopausal patients (Table 3). In premenopausal patients, values for body image and fatigue intensity were inferior at baseline, while postmenopausal patients reported inferior values for sexual functioning and enjoyment as well as upset by hair loss (Table 3).

**Table 3:** QoL and symptom severity at start of treatment (T0)

Scale	Range	Premenopausal (*n* = 251)		Postmenopausal (*n* = 478)	
		*n*	Mean ± SD	*n*	Mean ± SD
FACT-G^a^					
FACT-G global score	0–108	233	75.5 ± 15.40	426	75.8 ± 15.89
Physical well-being	0–28	236	18.4 ± 6.28	436	18.9 ± 6.40
Social/family well-being	0–28	235	23.1 ± 4.68	429	22.9 ± 4.81
Emotional well-being	0–24	237	17.9 ± 4.09	436	17.9 ± 4.45
Functional well-being	0–28	237	15.9 ± 5.89	436	15.9 ± 5.87
FACT-Taxane^a^					
FACT-Taxane subscale	0–64	231	58.7 ± 5.59	434	57.0 ± 7.82
FACT-ES^a^					
Endocrine Symptom Subscale-18	0–72	235	59.8 ± 7.45	436	60.2 ± 8.15
EORTC QLQ-BR23^a^					
Body image	0–100	235	64.3 ± 30.40	436	70.0 ± 30.05
Future perspective	0–100	232	47.0 ± 31.33	434	44.5 ± 31.75
Sexual functioning	0–100	226	29.9 ± 28.92	377	17.8 ± 25.06
Sexual enjoyment*	0–100	110	77.6 ± 25.98	99	63.3 ± 24.51
EORTC QLQ-BR23^b^					
Systemic therapy side effects	0–100	236	41.7 ± 21.47	437	39.9 ± 21.34
Breast symptoms	0–100	235	24.0 ± 22.86	434	22.1 ± 20.84
Arm symptoms	0–100	235	22.3 ± 22.44	435	21.5 ± 22.19
Upset by hair loss	0–100	225	39.3 ± 40.51	423	44.6 ± 43.38
Brief fatigue inventory^b^					
BFI total score	0–10	225	3.2 ± 2.32	416	3.0 ± 2.31
Fatigue intensity	0–10	224	4.2 ± 2.37	402	3.6 ± 2.37
Fatigue interference	0––10	225	2.8 ± 2.52	417	2.7 ± 2.41
HADS^b^					
HADS total score	0–42	229	11.2 ± 6.69	419	11.5 ± 6.95
Anxiety	0–21	232	5.8 ± 3.34	424	5.9 ± 3.57
Depression	0–21	231	5.3 ± 3.97	423	5.6 ± 3.93

FACT-G global score: PWB + SWB + EWB + FWB

*BFI* Brief Fatigue Inventory, *HADS* hospital anxiety and depression scale, *SD* standard deviation

*If sexually active

^a^High scores indicate high quality of life/low symptom severity

^b^High scores indicate high symptom severity

### Mean change in QoL from start of treatment (T0)

**Fig. 2:**
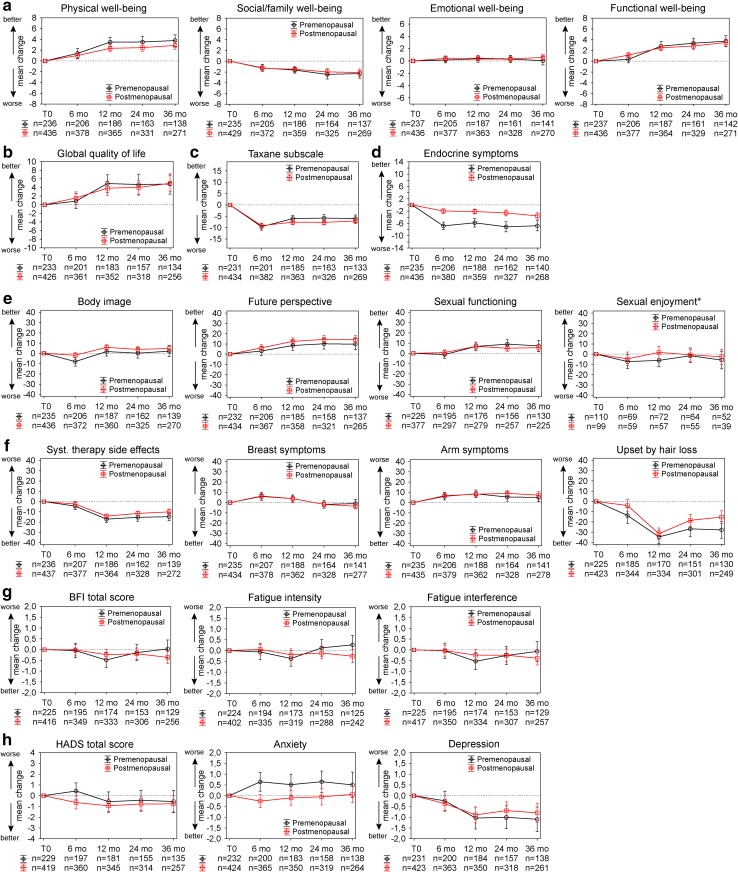
Mean change in quality of life over 3 years. Shown is the mean change from start of therapy (T0) until 6, 12, 24 and 36 months later. Error bars represent the 95% confidence interval at each time point. Higher values indicate an improved quality of life in **a** FACT-G subscales and **b** FACT-G total score, **c** FACT-Taxane subscale, **d** FACT-ES endocrine symptoms subscale and **e** EORTC QLQ-BR23 functional scales. Higher values indicate a worsening of symptom severity in **f** EORTC QLQ-BR23 symptom scales, **g** BFI total score and subscales and **h** HADS total score and subscales. *If sexually active. *BFI* Brief Fatigue Index, *HADS* Hospital Anxiety and Depression Scale, *mo* months, *syst* systemic

Looking at the mean change from start of treatment for the FACT-G questionnaire, there was improvement in physical and functional well-being and a decrease in social/family well-being (Fig. 2a). However, it needs to be mentioned that the mean score for social/family well-being at T0 was rather high. The mean change for the FACT-G total score showed an increased global QoL from start of treatment until 3 years later (Fig. 2b). The FACT-Taxane subscale, however, showed a persistent increase in neurotoxicity symptoms (Fig. 2c). The mean change for the endocrine symptom subscale showed an increase in symptoms, markedly more so for premenopausal patients (Fig. 2d). The mean change of the EORTC QLQ-BR23 functional scales (Fig. 2e) and symptom scales (Fig. 2f) showed no differences according to the menopausal status of the patients. The future perspective increased while the side effects of systemic treatment decreased. While the mean change in the fatigue scores showed a slight decline for the postmenopausal patients, the premenopausal patients seemed to recover from an initial slight decrease (Fig. 2g). While the mean change in the HADS depression subscale showed a decrease in depression over time, the mean change in the anxiety subscale revealed a lingering increase in anxiety in premenopausal patients (Fig. 2h).

### Clinical meaningful change in QoL

**Fig. 3:**
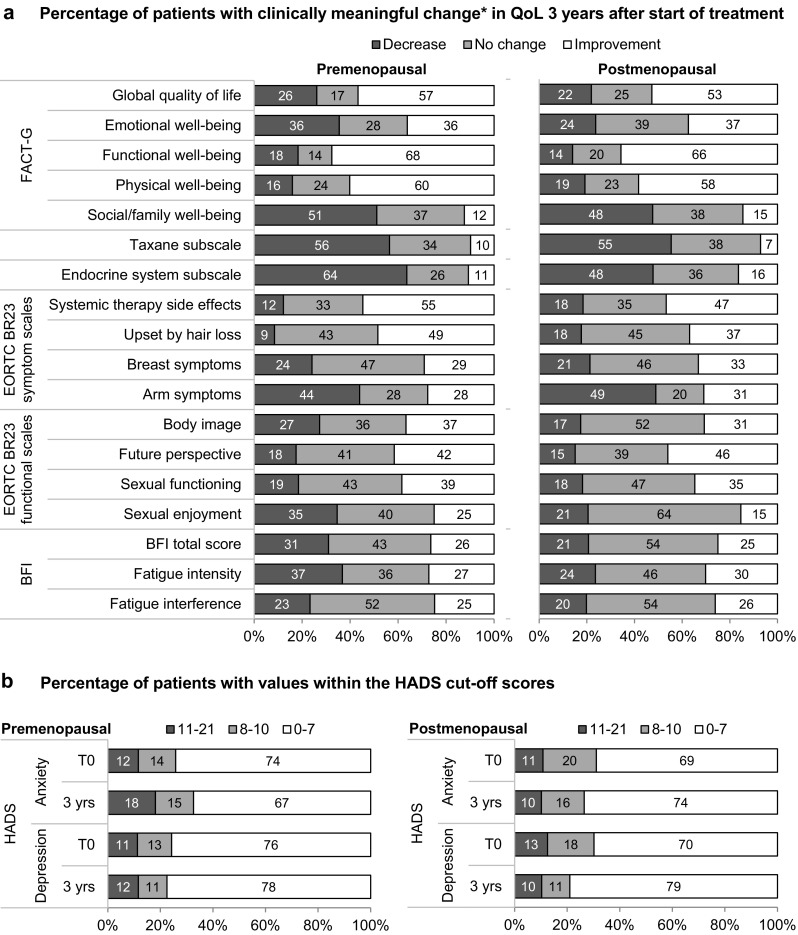
Clinically meaningful changes in QoL 3 years after start of treatment. **a** Percentage of patients with clinically meaningful change in each QoL score 3 years after start of treatment in comparison to the values at T0. All patients who returned the questionnaires at start of treatment and 3 years later were included in this analysis, the respective *n* corresponds to the number of patients indicated at the 36 months of time point in Fig. 2. *Minimal important difference: 10 points for EORTC QLQ-BR23 scales, 4 points for FACT-G total scale, 2 points for FACT-G subscales, ½ of the baseline standard deviation for the Taxane subscale (3 points for pre- and 4 points for postmenopausal patients), for the Endocrine symptoms subscale (4 points), the BFI total score (1.2 points), the Fatigue intensity scale (1.2 points) and the Fatigue interference scale (1.3 points for the pre- and 1.2 points for the postmenopausal patients). **b** Percentage of patients within the HADS categories at start of treatment (T0) and 3 years later. *yrs* years

The proportion of patients with clinically meaningful changes in the different QoL domains from start of treatment to 3 years later is shown in Fig. 3. The definition of the respective minimal important difference is described in the “Statistical Analysis” section. The global QoL as assessed with the FACT-G improved considerably for approximately half of the patients (Fig. 3a). 36% of the premenopausal and 37% of the postmenopausal patients reported a meaningful increase in emotional well-being; in contrast, 36% of the premenopausal and 24% of the postmenopausal patients reported a meaningful decrease. The scales with the worst deterioration 3 years after start of treatment were social/family well-being, the taxane subscale and the endocrine symptom subscale. Roughly half of the patients reported a significantly worse social/family well-being, regardless of the menopausal status. Neurotoxicity symptoms as assessed with the taxane subscale, worsened considerably for 55–56% of the patients. 64% of the premenopausal patients (48% of the postmenopausal patients) reported a clinically meaningful worsening of endocrine symptoms. Regarding the side effects of systemic treatment, 55% of the premenopausal (47% of the postmenopausal) patients reported clinically meaningful improvement. 44–49% of the patients reported an increase in arm symptoms and 27% of the premenopausal and 17% of the postmenopausal patients reported a decrease in body image. 37% of the premenopausal (24% of the postmenopausal) patients reported a worsening in fatigue intensity 3 years after start of treatment.

Looking at anxiety and depression as assessed with the HADS (Fig. 3b), there was an increase in the proportion of premenopausal patients with anxiety (14% doubtful and 12% definite cases at T0 vs. 15% doubtful and 18% definite cases 3 years later) and no change in the proportion of premenopausal patients with depression (Fig. 3b). Contrarily, the proportion of postmenopausal patients with anxiety decreased slightly and the proportion with depression decreased considerably (18% doubtful and 13% definite cases at T0 vs. 11% doubtful and 10% definite cases 3 years later).

Because most chemotherapies are finished 6 months after start of treatment (T1), we took a closer look at the clinical meaningful change from this time point until 3 years later. The mean values at T1 are shown in Table S1, and the percentages of patients with clinically meaningful changes in QoL in this period are presented in Figure S1. The results show that 59% premenopausal and 55% postmenopausal patients report a significant improvement in functional well-being from end of chemotherapy until 3 years later. Likewise, 55% premenopausal and 45% postmenopausal patients report a meaningful improvement in physical well-being in this period (Figure S1). Looking at social/family well-being, 25% premenopausal and 19% postmenopausal patients reported a significant improvement while 42% premenopausal and 39% postmenopausal reported a significant deterioration. A total of 49% premenopausal and 38% postmenopausal patients reported a meaningful improvement in neurotoxicity (Taxane subscale), while 18% premenopausal and 27% postmenopausal patients reported a meaningful deterioration. However, the mean FACT-Taxane scores at T1 were inferior to the mean scores at T0 (premenopausal 49.7 vs. 59.8; postmenopausal 47.9 vs. 60.2, Table 3 and S1). Similar tendencies were reported for the endocrine symptom subscale: 33% premenopausal and 22% postmenopausal patients reported a significant improvement, 30% premenopausal and 32% postmenopausal patients a significant deterioration after end of chemotherapy. These initial deteriorations and subsequent slight improvement/stabilisation are also visible in the mean change graph (Fig. 2).

Of note, patients reporting poor QoL at T1 and no improvement 3 years later appear in the “no change” category, just like the patients reporting good QoL at both time points.

## Discussion

This first interim analysis of the MaLife project aims at a comprehensive assessment of the QoL of patients with early breast cancer from start of treatment until 3 years later, with a special focus on the menopausal status. Regardless of the menopausal status, a high percentage of patients reported substantial improvements in global quality of life, especially in functional and physical well-being as well as for side effects of systemic treatment and depression. However, approximately half of all patients reported a distinct decrease in social/family well-being and a worsening of arm symptoms, endocrine symptoms and neurotoxicity symptoms. The presented data may help the treating physicians to discuss the situation with their patient—by looking at the clinically meaningful changed QoL scores both at start of treatment and after end of chemotherapy. Our data show that pre- and postmenopausal patients have differing needs in follow-up care. A higher percentage of premenopausal patients report worsening in body image, endocrine symptoms, fatigue intensity and anxiety 3 years after start of treatment. Especially for these young patients, mostly focused on career and caring for young children, improvement in treatment and follow-up care is urgently needed.

As expected, not all initially participating patients sent back the subsequent questionnaires, representing one potential limitation of this project. However, the return rate of the questionnaires was exceptionally high, strengthening the generalisability of our data. Due to the high proportion of patients receiving chemotherapy, the results from our cohort may not be generalised to patients receiving endocrine therapy, because the symptoms might differ. Strengths of this project are the prospective, longitudinal data collection and the participation of oncologists from all over Germany recruiting a large, representative study cohort.

QoL plays an important role in recovery—especially after an incisive diagnosis such as breast cancer. QoL assessments have been shown to improve the communication between physician and patient and encourage shared decision making [reviewed in 6]. The pre- and postmenopausal patients with early breast cancer in the MaLife cohort displayed a comparable distribution of receptor status and tumour stage. Additionally, approximately the same proportion of pre- and postmenopausal patients received radiotherapy and breast-conserving surgery. Both pre- and postmenopausal women reported an increase in global QoL over time, in contrast to previous reports stating that older patients adjust more easily to their breast cancer diagnosis than younger women [22, 37, 38].

Comparing our data on the mean FACT-G global score 3 years after start of systemic therapy (81.6 for pre- and 81.1 for postmenopausal patients) with reference data for the general population, American women reported a similar QoL (80.1), while Austrian women reported a slightly better global QoL (85.5) than the MaLife cohort [31, 39, 40]. Regardless of the menopausal status, the social/family well-being decreased steadily—approximately half of the patients reported a clinical meaningful worsening 3 years after start of treatment. Of note, the mean scores at baseline for pre- and postmenopausal patients (23.1 and 22.9) were higher than the scores reported by women of the general population (20.4, Austria and 19.1, United States) or women with breast cancer (18.3, Austria) [31, 39, 40]. Nevertheless, the constant decrease warrants improvements in follow-up care addressing social well-being.

The younger, premenopausal patients (median age 45 years) more frequently reported a clinically relevant worsening of fatigue intensity, body image and endocrine symptoms 3 years after start of treatment. Furthermore, 15% of the premenopausal patients classified as “doubtful cases” of anxiety and 18% as “definite cases” of anxiety 3 years after start of treatment. It has been published before that younger women report greater changes in body image, sexuality and mood, but also worse emotional and social functioning [22, 32, 41, 42]. This is probably due to treatment-related menopause, causing menopausal symptoms and infertility with a distinct negative impact on QoL [42, 43]. However, other aspects influencing the QoL in younger women are the circumstances—until diagnosis of breast cancer, younger women mostly pursue their career and are engaged in child-rearing activities. Treatment time, fatigue, pain and fear of relapse influence their daily life profoundly, although natural ageing processes must also be considered. Another aspect that needs to be addressed in follow-up care is neurotoxicity. The main symptom of neurotoxicity is peripheral neuropathy, associated with paraesthesia and numbness of fingers and toes, which is known to persist for years [15, 44–46]. Indeed, 55–56% of the patients in the MaLife cohort reported a clinically meaningful worsening of taxane-related toxicity 3 years after start of treatment, confirming the persistence of symptoms. However, after end of chemotherapy until 3 years later, 49% premenopausal and 38% postmenopausal patients reported a significant improvement in neurotoxicity symptoms. Taxanes are widely used in the adjuvant setting, also in patients with HR-positive tumours, despite current guidelines recommending only endocrine treatment for HR-positive breast cancer [8, 9]. There are still no recommendations for prevention and treatment of peripheral neuropathy [reviewed in 47] and research in that direction is of clinical importance.

## Conclusion

Our comprehensive assessment of QoL over the course of 3 years in women with early breast cancer treated in routine care in Germany highlights the areas requiring special attention in treatment decision making and follow-up care. Even 3 years after start of treatment, approximately half of the patients report a clinically significant decrease in social/family well-being, as well as a worsening of arm symptoms, neurotoxicity and endocrine symptoms. Younger, premenopausal patients more frequently report worsening in emotional well-being, anxiety, body image and endocrine symptoms in comparison to the values at baseline. It is of high interest for all physicians to discuss these topics with their patients and indicate those areas requiring special attention during follow-up care.

## Electronic supplementary material

Below is the link to the electronic supplementary material.

Supplementary material 1 (PDF 130 KB)
