# Supplementary material for: Quality of life in pre- and postmenopausal patients with early breast cancer: a comprehensive analysis from the prospective MaLife project
Source: Breast Cancer Res Treat. 2019 Mar 13;175(3):701–12. doi: 10.1007/s10549-019-05197-w (PMC6534521; doi:10.1007/s10549-019-05197-w)
Supplement: Supplementary file 1 — Supplementary material 1 (PDF 130 KB) [file 10549_2019_5197_MOESM1_ESM.pdf]

Table S1. QoL and symptom severity 6 months after start of treatment (T1)

		Premenopausal		Postmenopausal	
**Scale**	**Range**	**n**	**Mean ± SD**	**n**	**Mean ± SD**
**FACT-G[a]**					
FACT-G global score	0 – 108	214	77.2 ± 15.16	400	77.4 ± 16.56
Physical well-being	0 – 28	218	20.4 ± 5.24	409	20.1 ± 5.64
Social/family well-being	0 – 28	217	21.9 ± 5.09	408	21.5 ± 5.40
Emotional well-being	0 – 24	216	18.3 ± 4.08	410	18.4 ± 4.47
Functional well-being	0 – 28	217	16.5 ± 5.62	410	17.2 ± 5.81
**FACT-Taxane[a]**					
FACT-Taxane subscale	0 – 64	216	49.7 ± 12.53	415	47.9 ± 12.69
**FACT-ES[a]**					
Endocrine Symptom Subscale-18	0 – 72	218	53.7 ± 9.52	413	58.3 ± 9.26
**EORTC QLQ-BR23[a]**					
Body image	0 – 100	217	57.6 ± 33.19	405	68.4 ± 29.89
Future perspective	0 – 100	218	50.8 ± 32.03	401	51.4 ± 32.81
Sexual functioning	0 – 100	211	27.6 ± 25.97	344	19.3 ± 24.83
Sexual enjoyment *	0 – 100	96	67.0 ± 27.57	101	61.1 ± 26.70
**EORTC QLQ-BR23[b]**					
Systemic therapy side effects	0 – 100	218	36.0 ± 22.17	409	37.7 ± 21.40
Breast symptoms	0 – 100	219	29.4 ± 25.75	412	28.9 ± 26.56
Arm symptoms	0 – 100	218	27.9 ± 25.44	412	28.9 ± 25.81
Upset by hair loss	0 – 100	203	25.9 ± 40.56	389	40.5 ± 43.01
**Brief fatigue inventory[b]**					
BFI total score	0 – 10	214	3.0 ± 2.10	394	2.9 ± 2.25
Fatigue intensity	0 – 10	214	3.9 ± 2.26	391	3.6 ± 2.38
Fatigue interference	0 – 10	214	2.6 ± 2.24	394	2.6 ± 2.34
**HADS[b]**					
HADS total score	0 – 42	214	11.5 ± 7.06	406	11.0 ± 7.64
Anxiety	0 – 21	214	6.3 ± 3.64	408	5.7 ± 3.93
Depression	0 – 21	216	5.2 ± 3.98	406	5.2 ± 4.19

[a] High scores indicate high quality of life/low symptom severity.
[b] High scores indicate high symptom severity.
* If sexually active.
FACT-G global score: PWB+SWB+EWB+FWB.
Abbreviations: BFI, Brief Fatigue Inventory; HADS, hospital anxiety and depression scale; SD, standard deviation.

Figure S1. Percentage of patients with clinically meaningful change* in QoL T1 → T4

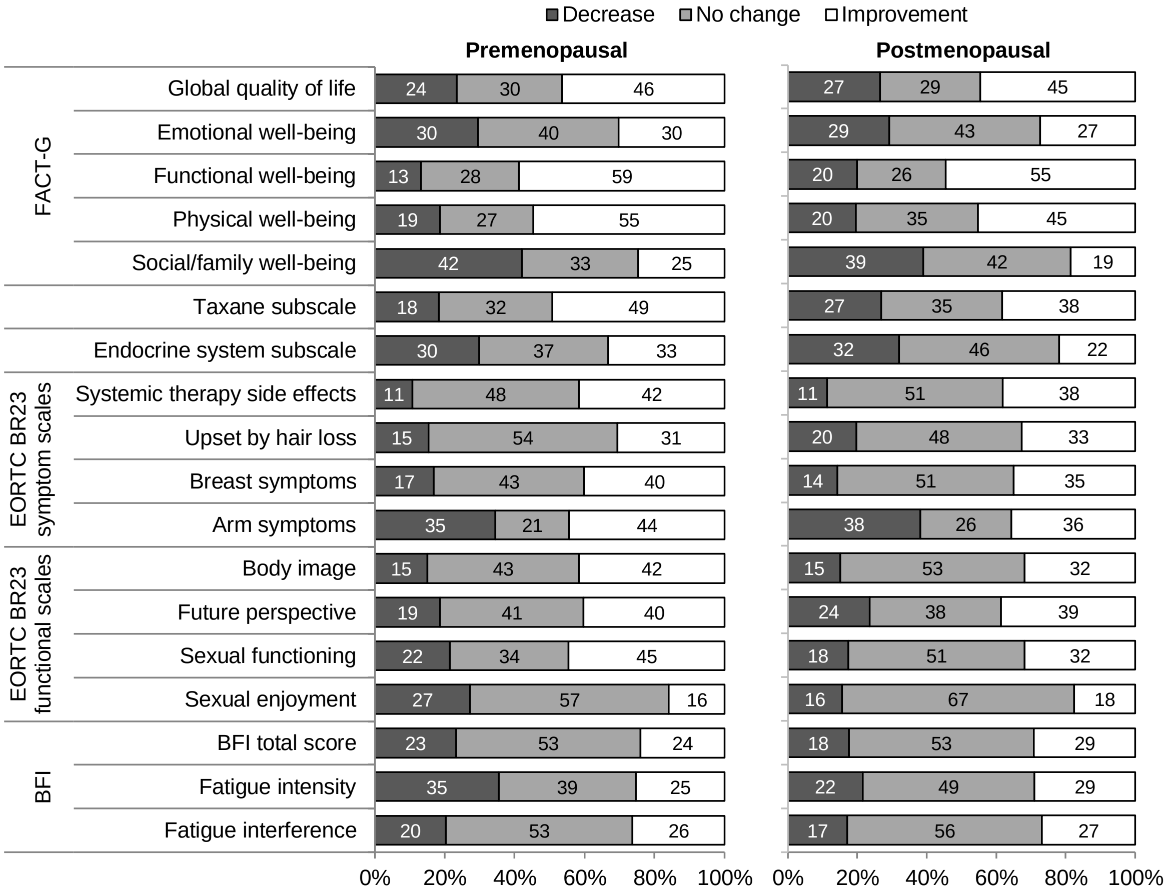

Fig. S1 Clinically meaningful changes in QoL from 6 months until 3 years after start of treatment
Percentage of patients with clinically meaningful change in each QoL score 3 years after start of treatment in comparison to the values at T1. Of note, patients reporting poor QoL at T1 and no improvement 3 years later appear in the "no change" category, just like the patients reporting good QoL at both time points.
*Minimal important difference: 10 points for EORTC QLQ-BR23 scales, 4 points for FACT-G total scale, 2 points for FACT-G subscales, ½ of the baseline standard deviation for: the Taxane subscale (3 points for pre- and 4 points for postmenopausal patients), the Endocrine symptoms subscale (4 points), the BFI total score (1.2 points), the Fatigue intensity scale (1.2 points) and the Fatigue interference scale (1.3 points for the pre- and 1.2 points for the postmenopausal patients).
